# Demographic and Socioeconomic Disparities in Congenital Hand Difference Surgery

**DOI:** 10.1007/s12178-026-10018-x

**Published:** 2026-03-17

**Authors:** Erik C. B. King, Quinci Howard, Elizabeth Santucci

**Affiliations:** 1 Division of Orthopaedic Surgery and Sports Medicine, Ann and Robert H. Lurie Children’s Hospital of Chicago, 225 E. Chicago Ave., Chicago, IL 60611 USA; 2 Department of Orthopaedic Surgery, Northwestern University Feinberg School of Medicine, Chicago, IL USA; 3 Department of Orthopaedic Surgery, Franciscan Health Olympia Fields Residency Program, Chicago, IL USA

**Keywords:** Congenital hand difference, Congenital hand surgery, Socioeconomic, Demographic, Disparities, Quality of life

## Abstract

**Purpose of Review:**

This article aims to identify and discuss the demographic and socioeconomic disparities that exist within the field of congenital hand difference surgery.

**Recent Findings:**

Current literature is somewhat limited regarding disparities within congenital hand difference surgery given the rarity in clinical practice and availability of data. Individuals with congenital hand differences have reported decreased upper extremity functional scores when compared to their peers. Specific demographic backgrounds, geographic locations, and socioeconomic status have been associated with lower quality of life, psychosocial well-being, and functional outcome scores. The orthopaedic surgeon’s role as a healthcare team facilitator and communicator is of high importance during the care of these patients and their families.

**Summary:**

Gender, racial, and socioeconomic disparities exist within the field of congenital hand difference surgery. A deliberate cognizance of these inequities during the evaluation and management of individuals with congenital hand differences is necessary. Further research and efforts aimed at mitigating the negative functional and psychosocial impact caused by these disparities should be continued.

## Introduction

Congenital hand differences are inherited or spontaneous anomalies affecting the hand that are, by definition, present at birth. These may occur in up to 23 per 10,000 live births and are frequently diagnosed in the neonatal or infantile age [1]. Updated Oberg, Manske, and Tonkin (OMT) classifications encompass four categories of congenital hand differences including malformations, deformations, dysplasias, and syndromes [2]. Congenital hand differences exhibit a unique challenge for most patients, families, and orthopaedic surgeons whereby treatment objectives emphasize function, physical appearance, and psychosocial well-being. Past literature has focused not only on early identification and the importance of prompt intervention of congenital hand differences, but has also sought to identify demographic and socioeconomic disparities that exist within the field of congenital hand difference surgery. It has been demonstrated throughout the field of congenital orthopaedic disorders that inequalities exist due to insurance status, income, race, place of residence and parental involvement, leading to limited access to care and delay in clinical presentation, and therefore, delay in treatment [3]. There may also be a higher prevalence of congenital hand differences within children experiencing socioeconomic deprivation [4]. Socioeconomic deprivation has been defined by level of income, employment, education, health, access to services, crime, and housing status [4].

Children and their families travel on average a distance of 66 miles to the nearest children’s hospital for evaluation and management of congenital hand differences. Additional travel typically occurs if treatment at high-volume hospitals is sought [5]. Therefore, those with limited transportation or parental unavailability, including single-parent households, may be significantly disadvantaged even before receiving their initial diagnosis or treatment plan. The need to attend multiple follow-up visits further intensifies these barriers. Typically, the increased financial costs are not anticipated prior to birth, and affected individuals may face a lifetime of medical costs and disability. Consideration of financial impact must be included in the discussion of these disparities.

The median total payments for common congenital hand difference surgical procedures averages $3,763 with a median out-of-pocket payment of $544, with some reports of total hospital billed charges exceeding $17,000 [5, 6]. It should be emphasized that congenital hand differences require a dedicated comprehensive management approach and may include physical or occupational therapy, routine follow up appointments, imaging, secondary procedures, prosthetic devices, psychologic care, travel expenses and other family-related costs.

This article aims to review existing literature regarding the current state of congenital hand difference surgery in an effort to identify and analyze the impact of demographic and socioeconomic status on outcomes including quality of life, psychosocial well-being, and trends within surgical care. Given the low prevalence of these differences, we will also discuss the limitations that exist within our current knowledge of congenital hand difference care disparities and identify areas for future research.

## Methods

### Study Selection, Eligibility Criteria, and Search Strategy 

A comprehensive literature search was conducted through the following databases: PubMed, Medline (EBSCOhost), Embase, Web of Science. In an effort to identify potential relevant articles the search was conducted using multiple keywords. The first set of keywords identified literature discussing specific patient populations and medical conditions using the terms “congenital hand difference”, “congenital upper limb difference”, “congenital hand anomaly”, “congenital hand surgery”. The second set of keywords recognized disparity terms including “disparities,” “inequity,” “inequities,” “socioeconomic,” “race,” “ethnicity,” “insurance,” “social determinants of health,” “access to care,” “outcomes” along with demographic and socioeconomic terms such as “access” or “demographic” or “socioeconomic” or “income” or “education” or “neighborhood” or “gender”. The literature search was performed on December 1, 2025. Studies and articles that were published within the date range of ‘January 10, 2010 to present’ were included.

### Data Extraction, Synthesis, and Statistical Analysis 

Seventeen articles were included based on the criteria and search strategy listed. The existing literature was evaluated and analyzed for information in regard to demographic and socioeconomic status, and the impact of specific factors on patients and families affected by congenital hand differences. Additional included articles evaluated the current structure of registry data, surgical timing, intervention, and other factors that may play a role in the outcomes of nonsurgical and surgical management of congenital hand differences. Existing literature and key points were extrapolated and are discussed throughout the article.

## Results

### Quality of Life

Congenital hand differences can have a significant impact on quality of life, despite advances in surgical interventions. In addition, some congenital hand differences are associated with genetic or syndromic conditions. Patients with medical comorbidities have been shown to have poorer outcomes [7, 8]. In the discussion of functional outcomes and quality of life of affected individuals, Patient-Reported Outcomes Measure Information System (PROMIS) and Pediatric Outcomes Data Collection Instrument (PODCI) are commonly referenced. Within PROMIS, the most useful domains are upper extremity function, pain, depression, anxiety, peer relations, and within PODCI, the domains of upper extremity function, mobility, physical function, comfort, happiness, satisfaction, and expectations are used. For all patient demographics, individuals with congenital upper limb differences have been shown to score lowest in the category of upper extremity function [8, 9]. In determining the impact of disparities on quality of life among individuals with congenital hand differences, both geographic and demographic factors have been studied. In a study by Daley et al., 260 patients with congenital upper limb differences were characterized by age, gender/sex, race, ethnicity, education, additional medical conditions, area deprivation index (ADI) and hand dominance. Using the PROMIS domains, parents from the Midwest geographic region (Missouri, Ohio, Minnesota), reported higher perceived upper extremity function of their children than parents in the West geographic region (Washington, California, Utah) (*p* < 0.05) [7]. Additionally, Black children were found to have significantly lower upper extremity functional scores than their White or Asian counterparts [7].

### Psychosocial Impact

Overall well-being is an integral part of quality of life, and hence, similar outcomes have been shown in studies evaluating both quality of life and the psychosocial impact of congenital hand differences on patients and their families. In a study by Wilks et al., upper extremity functional scores were shown to be positively correlated with psychosocial well-being, emphasizing the importance of function on overall health [10]. Discrepancy within these scores exists when considering gender and race. As evidenced by Daley et al., Black children with congenital upper limb differences have lower reported upper extremity functional scores and higher rates of pain, depression, and anxiety than White or Asian children [7]. Well-being scores of families of individuals with congenital hand differences were in general lower than the population norm but were not associated with disease severity. Within this population, females were specifically noted to have reduced well-being scores [11].

A caveat should be made that our understanding of the relationship is incomplete, evidenced by the fact that some patients with decreased upper extremity functional scores have been found to have normal psychosocial scores [8]. Clearly, the psychosocial impact of these conditions is multifactorial. The unique nature of congenital hand differences should be considered, and available resources and education should be tailored to patients and families who are managing and living with these differences. Miller et al. determined that among parents surveyed regarding the resources available to them, 68% of parents wanted improved access to psychologic support [11].

### Surgical Care and Timing

Early surgical intervention of congenital hand differences is presumed to lead to advantageous outcomes compared to delayed surgery. However, guidelines for appropriate surgical timing are controversial and must be decided in the context of the individual child’s health and development [12]. A retrospective study on correction of radial club hand revealed that surgical correction to be effective and safe as early as 10 months of age [13]. Certain demographic groups, such as those of female gender and children of Hispanic and Black race, have been documented as at-risk groups for undergoing late release of congenital hand differences [14].

When specifically discussing the management of syndactyly, the general consensus for management includes surgical release or correction before “school age”, although this is an undefined age. Recommendations for age at corrective surgery range anywhere from three to 24 months depending on the study and location of the anomaly. Most hand surgeons agree that it is beneficial to perform syndactyly surgical release prior to the age where fine motor skills are crucial, defined by Slevin et al. as before the age of two. In a retrospective review of 2,280 children who underwent syndactyly release, the overall mean age at syndactyly release was 3.6 years old. The data on the age at release was then divided into early (younger than five years old) and late (greater than or equal to five years old). The average age of children undergoing release in the early group was found to be much lower, at 2.5 years old. Late release was more commonly seen in female (37.9% vs. 30.1%, *p* = 0.002), Hispanic (41.8% vs. 37.3%, *p* = 0.024), and Black (12.4% vs. 11.1%, *p* = 0.024) children [14]. Although the percentage differences are small, they may not be inconsequential. Delays in care can correlate with negative outcomes and poor functional results.

Socioeconomic deprivation also contributes to surgical timing. Chan et al. studied 259 cases of congenital hand differences. Patients were distributed among quintiles based on deprivation indicators, including income, education, employment, access to services, health, crime, and housing. Those in Quintile 1 were designated the “most deprived” group, and those in Quintile 5 were designated as the “least deprived”. They found both a higher prevalence of congenital hand differences and a higher percentage of children undergoing operative intervention within Quintile 1 when compared to all other quintiles (*p* = 0.001 and *p* = 0.003, respectively). Further, the children within Quintile 1 were referred for surgical intervention at a younger age than those in Quintile 5 (average age 1.5 vs. 4.4 years, *p* = 0.007) [4].

Numerous studies have shown that higher volume medical centers have better outcomes in performing complex reconstructive procedures [15–19]. Patients with a higher socioeconomic status are more likely to undergo surgical management of their congenital hand difference at an institution with higher case volume [5]. Patients living in underserved areas were significantly less likely to be treated at high-volume hospitals (9.9% vs. 26.5%, *p* < 0.001). Urban-dwelling, higher family income, and commercial insurance significantly increased the likelihood of being treated at a high-volume hospital (defined as a volume within the 80th percentile of total congenital hand surgery procedures over a ten-year period). Children treated at hospitals with above-median case volumes underwent corrective surgery at a significantly earlier age (14.2 months vs. 16.4 months, *p* < 0.001) [5]. The average distance required for travel to the nearest children’s hospital was noted to be 66 miles, with some patients able to travel an additional 60 miles for higher-volume care. White patients specifically were more likely to seek care at a high-volume hospital, even if it was at a greater distance. Treatment at a higher-volume care center was positively correlated with a younger age of corrective operation, decreased hospital admission, and lower billed medical charges. Specifically, those with above-median income and with commercial insurance were noted to have a lower charge-to-cost-ratio [5].

### Surgical Experience and Patient Education

Caregivers of individuals with congenital upper extremity differences report substantial financial impact [20]. When measured by the Impact on Family Scale (IOFS), financial impact is greater in families categorized as lower socioeconomic status, such as those with a household income of $20,000-$40,000, families with single adult caregivers, or receiving public insurance. Bilateral upper extremity involvement and additional musculoskeletal diagnoses were also noted to increase family impact, and distance to see the surgeon was correlated with an increased financial-related family impact score. Finally, IOFS scores inversely correlated with PROMIS scores within the upper extremity, mobility, sports, pain, happiness, and global domains establishing the relationship of family burden on functional outcomes [20].

Education and understanding of the patient’s condition can also play a role in the family’s experience, and health literacy is important to discuss when addressing health disparities. The American Medical Association recommends distributing medical education that is written at a sixth-grade level (age 11 years) or less. When evaluating the available patient education, most online resources for those with congenital hand differences were found to be written above the recommended reading level [21].

### Registry Data

The Congenital Upper Limb Differences (CoULD) registry is one of the primary multicenter registry databases for collecting information regarding congenital upper limb differences, inclusive of individuals with congenital hand differences. CoULD was founded in 2014 in an effort to improve the understanding of congenital upper limb differences among families and orthopaedic hand surgeons, and to enable a unified database to enhance the treatment of congenital upper limb differences throughout the country. The creators of the CoULD registry have sought to address the challenges associated with collecting quality functional assessments and identifying surgical outcomes of children with rare congenital upper limb conditions. The number of institutions providing high volume congenital upper limb care and surgery throughout the nation is particularly limited, lending to difficulty with powering research studies and conducting multicenter trials [22]. CoULD now encompasses data from ten institutions, and is mostly representative of these tertiary care centers [23]. The continued growth and use of this registry will ultimately help us identify, study, and ameliorate health disparity in the treatment of congenital hand differences.

## Discussion

Review of the published studies within congenital hand difference surgery illustrates some of the disparities found within different demographic and socioeconomic groups. Income, geographic area, gender, and race have been noted to be significant factors in disparate outcomes of individuals with congenital hand differences. The burden of additional medical conditions may be heightened in those with socioeconomic disparities, and therefore identifying this population may play a role in optimizing surgical outcomes.

Chan et al. identified a disparity in the overall prevalence of congenital hand differences between patients within the most socioeconomically deprived group (22 per 100,000 in the most deprived Quintile 1 versus 13 per 100,000 in the least deprived Quintile 5) and noted that individuals within Quintile 1 underwent operative treatment at a higher rate [4]. Their findings here were surprising, and it is unclear what led to the higher percentage of operative intervention among the most deprived quintile. The authors acknowledge that numerous confounding variables prevent a definitive conclusion. Their data was collected from a single tertiary center, and they posit that increased exposure to environmental factors may predispose to non-chromosomal congenital differences including proximity to pollutants, maternal occupation, and other factors leading to low birth weight. In addition, maternal screening protocols may have varied among the studied populations. More research to determine the prevalence of congenital hand differences among different socioeconomic groups is warranted.

Multiple studies support that those of higher socioeconomic status are more equipped to undergo potentially life-altering surgical management at an earlier age and more likely to be treated at a higher volume hospital, which may lead to improved outcomes and decreased financial burden among those with the highest income [5, 24, 25]. Individuals who are of lower socioeconomic status may therefore be further disadvantaged by delayed intervention and increased financial stress due to transportation barriers, medical costs, insurance coverage, parental availability, and hospital/provider access. Although the positive correlation between surgical outcomes and hospital volume has been well-documented in medical literature, whether this truly equates to improved clinical and functional outcomes specifically for congenital hand surgery has not been adequately studied.

Clinicians should carefully consider the psychosocial impact of congenital hand differences. As related to quality of life, both physical appearance and upper extremity function play a significant role in the self-esteem and confidence of developing children. This may be heightened in female patients. In light of lower well-being and mental health scores seen in children with congenital hand differences, increased access to psychologic support, especially at key transitions and childhood milestones, should be encouraged throughout all demographics [11]. Psychosocial impact varies by socioeconomic status and race, as Black children have increased rates of anxiety, depression, and pain. Wall et al. further identified that greater social deprivation reflected higher anxiety, depression, and lower peer relations [7, 26]. Therefore, socioeconomic status could be the true correlation.

Parents and caregivers are typically highly involved in the diagnosis and management of congenital hand differences, and several factors may contribute to the increased family burden, including accessible healthcare, financial assistance, and health literacy. A larger reported family burden is associated with poorer PROMIS-measured functional and psychological outcomes. It is generally accepted that increased communication between the physician and patient/family unit facilitates more positive outcomes. Therefore, emphasis should be placed on providing appropriate educational resources for patients and families commensurate with individual health literacy, and to promote open communication within the healthcare team. Lian et al. recognized when parents and/or families felt inclusive within the healthcare team that the greatest benefits were achieved from surgical intervention [27]. This inclusiveness is promoted when the caregivers of the children have greater understanding.

The published studies highlight the relevance of financial costs, test availability, time constraints, and distance of specialists [28]. It may not be feasible or suitable to travel for numerous appointments at specialized tertiary care facilities. Identification of individuals and families who face adverse social determinants of health may aid in providing tailored medical care, and thus reduce burdens on families and ultimately improve outcomes. Outreach clinics held in low-resourced communities may also be beneficial, and physician-patient communication can be enhanced through physicians who demonstrate cultural awareness and competency, knowledge of local current events, and appropriate patient geographic constraints selection, if travel to other areas is pursued [29].

Overall, the use of the CoULD registry has been helpful in identifying risk factors for congenital hand differences, decision-making algorithms for complex cases, and patient-reported outcomes to help guide treatment methods. This registry has created a much-needed central resource for managing these complex conditions nationwide and has enabled ongoing research efforts. While lauding the creation of the CoULD registry, there are some caveats. Vuillermin et al. identified a registry inclusion effect, by which the registry was initiated with a population of patients who had not yet undergone operative intervention for their congenital hand difference. An underrepresentation of operatively managed congenital hand differences, such as polydactyly, was noted within the first three years of registry initiation, which lessened as more patients were enrolled [23]. This registry may also capture later-presenting differences when compared to birth registries, a higher prevalence of rare conditions, and a disproportionate representation of certain conditions due to the long enrollment window and geographic area of the ten participating institutions [23]. This bias is important to consider when evaluating information from the early years of database creation and when new institutions are added to the registry in the future.

### Limitations

Our understanding of the demographic and socioeconomic disparities within the field of congenital hand difference surgery is limited by the low incidence of these conditions and the paucity of published studies on this topic. The rarity of these conditions makes it difficult to identify and generalize factors that may play a role in quality of life and psychosocial impact. The patients included in the CoULD registry and the basis of many studies originate from ten institutions in specific geographic areas and may not be generalizable to all populations with congenital hand differences. In particular, lower socioeconomic groups may be underrepresented, thus lessening the ability to identify disparities. Additionally, existing studies that are cross-sectional may not fully represent the impact of disparities over time.

## Conclusion

As has been described in many areas studied in health care, demographic and socioeconomic disparities are present in the delivery of care to individuals with congenital hand differences. Published studies have identified trends in surgical care, differing quality of life scores and greater family impact. Specific patient populations including female gender, Hispanic or Black race, and those living within certain geographic locations are at higher risk of poorer functional outcomes. The rarity of these conditions make determining exact causation challenging, and additional research should be performed in these areas to further study the disparities that exist. As surgical advances continue to evolve within the field of musculoskeletal care, it is important to identify patients with specific social determinants of health, such as low-income status or constrained access to healthcare services, in order to promote timely and affordable access to care, a positive patient and family experience, optimal functional outcomes, and the highest possible quality of life. Open communication between the orthopaedic surgeon and the patient-family unit involves discussing functional, physical, financial, and social expectations for those with congenital hand differences which may lead to decreased family burden and improved quality of life.

## Key References

• Chan CCH, Stirling PHC, Lam WL. “The impact of socioeconomic deprivation on congenital hand differences: A retrospective cohort study”. Hand surgery and rehabilitation (2468 − 1229), 41 (2), 265.
  - ◦ A retrospective study identifying the prevalence and differences within surgical care of congenital hand differences in individuals grouped by socioeconomic deprivation quintiles.
• Kalmar CL, Drolet BC. “Socioeconomic Disparities in Surgical Care for Congenital Hand Differences”. Hand (New York, N.Y.) (1558–9455), 19 (1), 104.
  - ◦ A retrospective study that evaluated the impact of socioeconomic and geographic factors on surgical care within the field of congenital hand difference surgery, identifying disparate access to care.
• Daley E, Peek K, Carlin, K, Samora J, Vuillermin C, Wall L, et al. “Effect of race and geography on patient- and parent-reported quality of life for children with congenital upper limb differences”. The Journal of Hand Surgery, 48(3), pp. 274–282. doi: 10.1016/j.jhsa.2022.10.018.
  - ◦ Using the CoULD registry, this study revealed differences within PROMIS scores between distinct geographic and racial groups.
• Slevin O, Beutel BG, Ohana N, Marascalchi B, Melamed E. “Factors Associated with Timing of Syndactyly Release in the United States”. The Journal of Hand Surgery Asian-Pacific volume (2424–8363), 27 (2), 294.
  - ◦ This study evaluated corrective surgery of syndactyly and investigated factors associated with operative timing, revealing that gender and race were associated with late release.

## Data Availability

No datasets were generated or analysed during the current study.
